# Age-Related Decline in Nrf2/ARE Signaling Is Associated with the Mitochondrial DNA Damage and Cognitive Impairments

**DOI:** 10.3390/ijms232315197

**Published:** 2022-12-02

**Authors:** Artem P. Gureev, Victoria G. Khorolskaya, Irina S. Sadovnikova, Ekaterina A. Shaforostova, Vadim R. Cherednichenko, Inna Y. Burakova, Egor Y. Plotnikov, Vasily N. Popov

**Affiliations:** 1 Department of Genetics, Cytology and Bioengineering, Voronezh State University, 394018 Voronezh, Russia; 2 Laboratory of Metagenomics and Food Biotechnology, Voronezh State University of Engineering Technologies, 394036 Voronezh, Russia; 3 A.N. Belozersky Institute of Physico-Chemical Biology, Lomonosov Moscow State University, 119992 Moscow, Russia

**Keywords:** aging, cognitive deficit, nuclear erythroid 2-related factor 2, mammalian target of rapamycin complex 1, brain-derived neurotrophic factor, mitochondrial DNA damage, Morris water maze

## Abstract

In this research, we compared the cognitive parameters of 2-, 7-, and 15-month-old mice, changes in mitochondrial DNA (mtDNA) integrity and expression of genes involved in the nuclear erythroid 2-related factor 2/antioxidant response element (Nrf2/ARE) signaling pathway. We showed an age-related decrease in the *Nfe2l2* expression in the cerebral cortex, not in the hippocampus. At the same time, we find an increase in the mtDNA copy number in the cerebral cortex, despite the lack of an increase in gene expression, which is involved in the mitochondrial biogenesis regulation. We suppose that increase in mtDNA content is associated with mitophagy downregulation. We supposed that mitophagy downregulation may be associated with an age-related increase in the mtDNA damage. In the hippocampus, we found a decrease in the *Bdnf* expression, which is involved in the pathways, which play an essential role in regulating long-term memory formation. We showed a deficit of working and reference memory in 15-month-old-mice in the water Morris maze, and a decrease in the exploratory behavior in the open field test. Cognitive impairments in 15-month-old mice correlated with a decrease in *Bdnf* expression in the hippocampus, *Nfe2l2* expression, and an increase in the number of mtDNA damage in the cerebral cortex. Thus, these signaling pathways may be perspective targets for pharmacological intervention to maintain mitochondrial quality control, neuronal plasticity, and prevent the development of age-related cognitive impairment.

## 1. Introduction

Aging is a physiological process associated with a decrease in the functionality of an organism’s cells. All cellular components require regeneration during life, which is associated with significant energy costs. Mitochondria, which play a key role in maintaining energy homeostasis and the metabolism of reactive oxygen species, also require constant turnover [1]. Despite being only 2% of body weight, the brain consumes about 20% of the energy received by the organism [2]. High energy requirements make this organ most susceptible to age-related changes. Impaired coordination of maintaining mitochondrial homeostasis leads to degenerative processes that may contribute to the pathogenesis of such a socially significant neurodegenerative diseases such as Alzheimer’s and Parkinson’s diseases [3]. Memory loss is a nonspecific symptom that occurs in many brain diseases, such as dementia and cognitive impairment. Common to all such pathologies is a pronounced decrease in neuronal plasticity, that is, the ability of neurons to change their functional properties and connections in response to changes in environmental conditions [4].

The development of pharmacological substances to support mitochondrial quality control and neuronal plasticity is an actual task for modern biological science. However, for this, it is necessary to find suitable targets for pharmacological agents. The Nrf2/ARE (nuclear erythroid 2-related factor 2/antioxidant response element) signaling pathway maintains mitochondrial homeostasis by induction of the expression of phase II detoxifying and oxidative-stress responsive genes and controls mitochondrial copy number by regulation of mitochondrial biogenesis and mitophagy, which is important for maintaining neuronal plasticity [5]. Mitochondrial DNA (mtDNA) is an optimal marker for the estimation of mitochondrial integrity. The mtDNA is located in the mitochondrial matrix, where the main production of reactive oxygen species (ROS) occurs [6]. The mtDNA is a histone-free nucleoid, which is less resistant to ROS-related damage compared with nuclear DNA [7]. The mtDNA does not have some of the repair mechanisms, such as nucleotide excision repair (NER), mismatch repair (MMR), homologous recombination, and non-homologous end joining (NHEJ) repair, which protect nuclear DNA from the accumulation of oxidative damage [8]. Probably, these features of mtDNA make it a relevant marker of age-related loss of integrity of mitochondria.

It was found that the Nrf2/ARE signal pathway affects the metabolism of some neurotransmitters, such as serotonin and dopamine [9]. Long-term memory formation requires strengthening neuronal connections, mostly in the hippocampus, associated with the structural rearrangement of neurons. The key role in the synthesis of proteins essential for these rearrangements belongs to mTORC1 (mammalian target of rapamycin complex 1) and the brain-derived neurotrophic factor (BDNF)/AKT1 pathway, which is involved in mTORC1 regulation [10]. Suppression of mTORC1 activity may impair synaptic plasticity and long-term memory [11].

There is evidence of a functional relationship between Nrf2 and mTORC1, which plays a key role in forming long-term memory [12]. We hypothesize that there is an impairment of functioning Nrf2/ARE and BDNF/AKT1/mTORC1 signal pathways with age. However, so far, there have been no studies investigating how interrelated these signaling pathways are in the process of age-related memory impairment. The aim of this research was to study the relationship between age-related cognitive changes in the expression of the Nrf2/ARE and BDNF/AKT1/mTORC1 signal pathways genes on 2-, 7- and 15-months old mice in the cerebral cortex and hippocampus. For the estimation of cognitive function, the Morris water maze (MWM) was performed. Age-related changes in anxiety and exploratory behavior were estimated using open-field and dark-light box (DLB) tests. The string test was used for the measurement of the strength and endurance of the mice of different ages. In parallel, we conducted an estimation of the copy number and integrity of mtDNA using long-range PCR, expression of genes involved in the mitochondrial biogenesis and inflammation processes.

## 2. Results

### 2.1. mtDNA Lesions in the Cerebral Cortex

**Figure 1:**
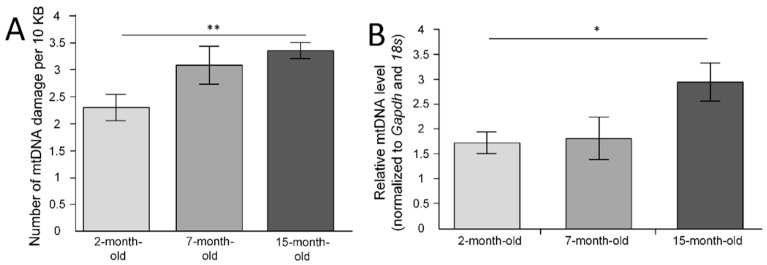
The age-related changes in mtDNA in the cerebral cortex in the three age groups of mice. (**A**) The mean number of damages through all studied fragments of mtDNA. (**B**) Normalized mtDNA level in the frontal cortex * *p* < 0.05, ** *p* < 0.01 differences between age groups were statistically significant according to the Mann–Whitney test. The 2-month-old mice (n = 9), 7-month-old mice (n = 9), 15-month-old mice (n = 9).

The number of mtDNA damage was measured in six fragments of mtDNA. During the analysis of the mean number of damages through all studied fragments of mtDNA, it should be noted that the 15-month-old mice had 46% more lesions than the 2-month-old mice (*p* < 0.01) (Figure 1A).

There was no age-related increase in the number of mtDNA damage in the fragments encoded *Nd5* gene (F(2, 24) = 2.1801, *p* = 0.13490) and *Nd6-CytB* genes (F(2, 24) = 1.1213, *p* = 0.34233). In the region of mtDNA, which encoded ribosomal RNA genes, there was an increase in the number of damages in the group of 7-month-old mice compared with 2-month-old mice (*p* < 0.01). A similar result was obtained for the region, encoded *Nd1-Nd2* genes (*p* < 0.05). For 15-month-old mice, the maximal number of mtDNA damage was noted in regions encoded *16s-Nd1* genes (*p* < 0.05 compared with 2-month-old mice) and RNA non-coding region D-loop (*p* < 0.001 compared with 2-month-old mice and *p* < 0.01 compared with 7-month-old mice) (Table 1).

### 2.2. mtDNA Copy Number in the Cerebral Cortex

We have shown age-related changes in the number of mtDNA copies (F(2, 24) = 3.7005, *p* = 0.03974). The normalized mean level of mtDNA in the group of 2-month-old mice was 1.7 ± 0.2. The mean level of mtDNA in the group of 7-month-old mice did not differ and was 1.8 ± 0.4. In the group of 15-month-old mice, the normalized level of mtDNA was 3 ± 0.4, which was statistically significantly higher compared with the 2-month-old mice (*p* < 0.05), but not with the 7-month-old mice (*p* = 0.094) (Figure 1B).

### 2.3. Gene Expression in the Cerebral Cortex

**Figure 2:**
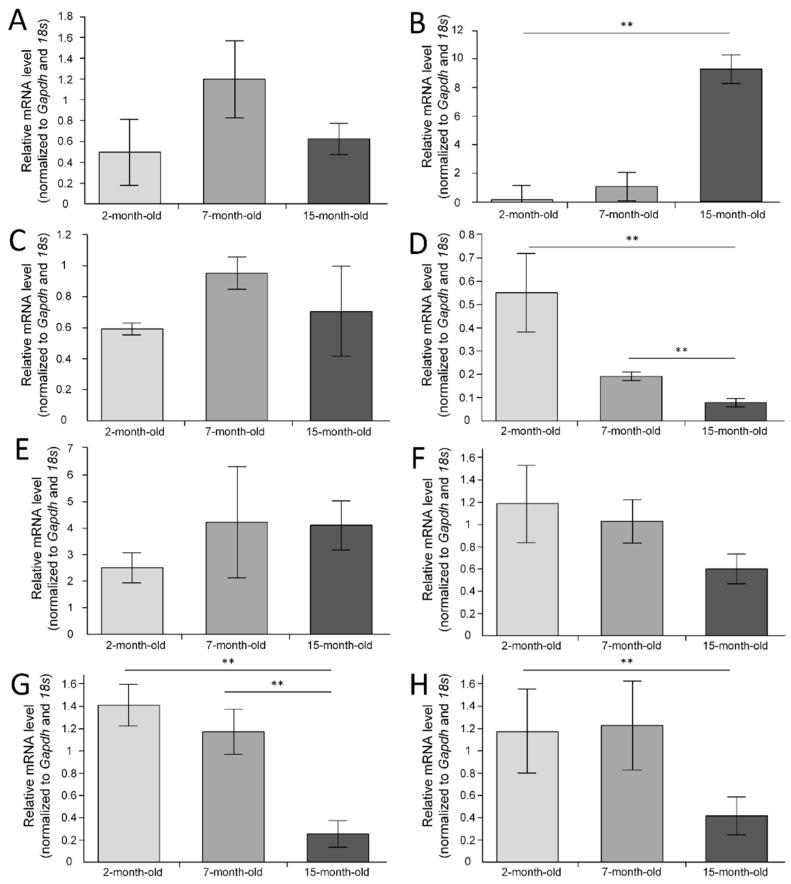
The normalized gene expression in the cerebral cortex: (**A**) *Bdnf*; (**B**). *Il1b*; (**C**) *Nrf1*; (**D**) *Nfe2l2*; (**E**) *Cox1*; (**F**) *Tfam*; (**G**) *Sod2;* (**H**) *p62*. ** *p* < 0.01 differences between age groups were statistically significant according to the Mann–Whitney test. The 2-month-old mice (n = 9), 7-month-old mice (n = 9), 15-month-old mice (n = 9).

*Bdnf* (brain-derived neurotrophic factor) expression did not differ between experimental groups (F(2, 24) = 1.6195, *p* = 0.21889) (Figure 2A). We observed *Il1b* (interleukin 1 beta) overexpression in 15-month-old mice, which was statistically significant compared with 2-month-old mice (*p* < 0.01), but compared with 7-month-old mice, the differences were statistically insignificant (*p* = 0.058) (Figure 2B). *Nrf1* (nuclear respiratory factor 1) expression did not differ between experimental groups (F(2, 24) = 1.0504, *p* = 0.36535) (Figure 2C). We found differences in the *Nfe2l2* (nuclear factor, erythroid-derived 2, like 2) expression (F(2, 24) = 6.2203, *p* = 0.00666). The level of expression of the *Nfe2l2* gene significantly decreased in 15-month-old mice compared to 2-month-old mice (*p* < 0.01) and compared with 7-month-old mice (*p* < 0.01) (Figure 2D). The level of *Cox1* (cytochrome c oxidase subunit 1) expression in the forebrain did not differ (F(2, 24) = 0.49427, *p* = 0.61609) (Figure 2E). We did not find differences in the expression of *Tfam* (transcription factor A, mitochondrial) (F(2, 24) = 1.6137, *p* = 0.22003) (Figure 2F). We found differences in the *Sod2* (superoxide dismutase 2) expression (F(2, 24) = 12.305, *p* = 0.00021). In the group of 15-month-old mice, *Sod2* expression was significantly reduced compared to 2-month-old mice (*p* < 0.001) and compared with 7-month-old mice (*p* < 0.01) (Figure 2G). We found differences in the *p62* expression (F(2, 24) = 5.0278, *p* = 0.01501). The level of expression of the *p62* gene significantly decreased in 15-month-old mice compared to 2-month-old mice (*p* < 0.05).

### 2.4. Gene Expression in the Hippocampus

**Figure 3:**
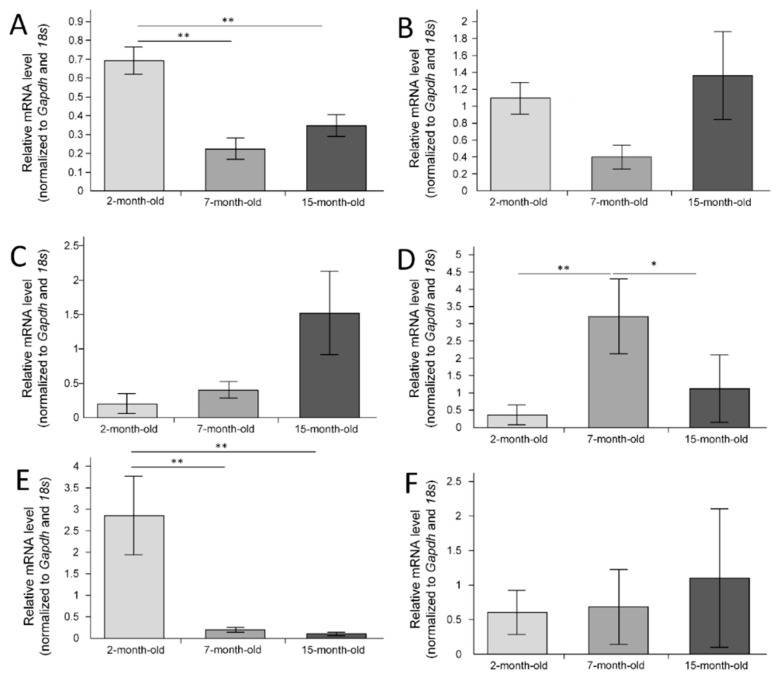
The normalized gene expression in the hippocampus: (**A**) *Akt1*; (**B**) *Mtor*; (**C**) *Nfe2l2*; (**D**) *Pten*; (**E**) *Bdnf*; (**F**) *Il1b*. The 2-month-old mice (n = 9), 7-month-old mice (n = 9), 15-month-old mice (n = 9). * *p* < 0.05; ** *p* < 0.01 differences between age groups were statistically significant according to the Mann–Whitney test.

We have shown age-related changes in the *Akt1* (serine/threonine kinase 1) expression (F(2, 24) = 14.960, *p* = 0.00006)*. Akt1* expression was maximal in 2-month-old mice and decreased in 7-month-old mice (*p* < 0.01) and in 15-month-old mice (*p* < 0.01) (Figure 3A). *Mtor* (mechanistic target of rapamycin kinase) expression did not differ in different age groups (F(2, 24) = 2.2781, *p* = 0.12421) (Figure 3B). ANOVA revealed an age-related difference in the *Nfe2l2* expression in the hippocampus, but post hoc tests did not show differences between groups despite a tendency to increase the expression with age (Figure 3C).

The maximum expression of *Pten* (phosphatase and tensin homolog) in 7-month-old mice was significantly higher than that in 2-month-old mice (*p* < 0.01) and 15-month-old mice (*p* < 0.05) (Figure 3D). Expression of the *Bdnf* gene was maximal in 2-month-old mice. Age-related decrease in the *Bdnf* expression was observed (F(2, 24) = 8.7934, *p* = 0.00136). The *Bdnf* expression level was reduced in 7-month-old mice (*p* < 0.05 compared with 2-month-old mice) and in 15-month-old mice (*p* < 0.01 compared with 2-month-old mice) (Figure 3E). Expression of the *Il1b* gene in the hippocampus did not change with age (F(2, 24) = 0.15217, *p* = 0.85966) (Figure 3F).

### 2.5. The Behavioral Changes

**Figure 4:**
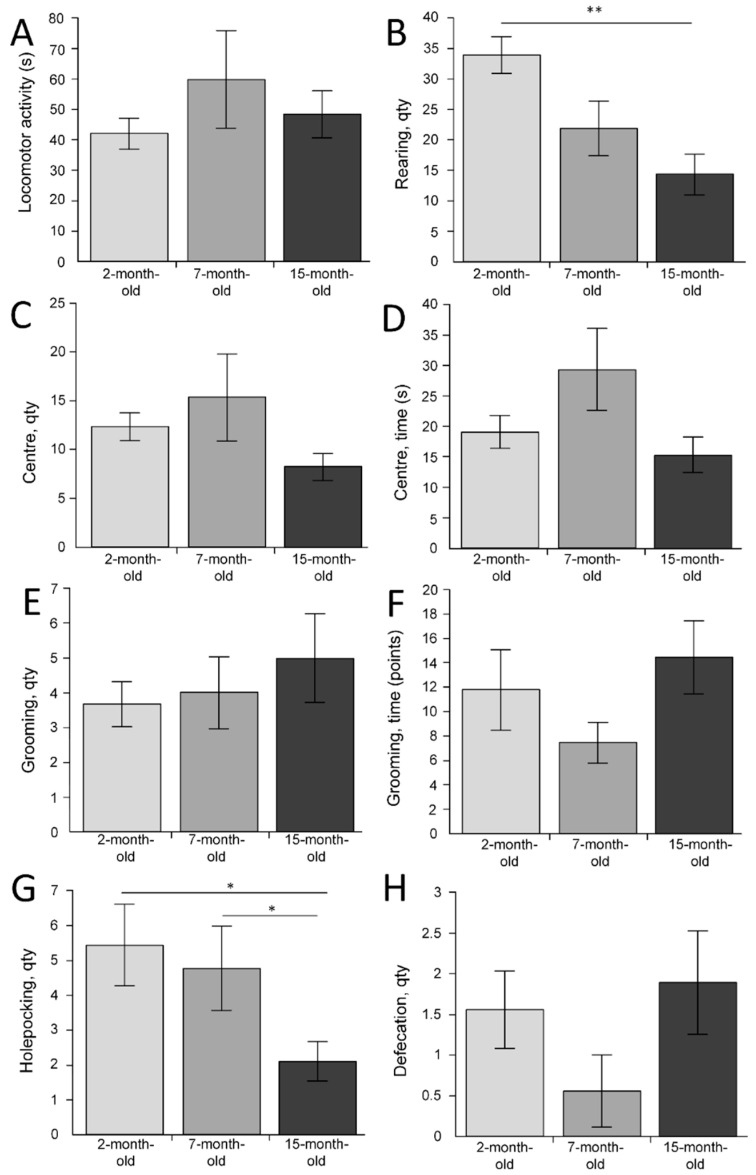
The results of the open field test for the three age groups. (**A**) Locomotor activity (s); (**B**) rearing acts (quantity); (**C**) entering to the center (quantity); (**D**) time in the center area (s); (**E**) grooming acts (quantity); (**F**) grooming time (s); (**G**) hole-poking acts (quantity); (**H**) defecation acts (quantity). * *p* < 0.05; ** *p* < 0.01 differences between age groups were statistically significant according to the Mann–Whitney test. The 2-month-old mice (n = 9), 7-month-old mice (n = 9), 15-month-old mice (n = 9).

The open field test showed that the duration of locomotor activity in mice did not differ between the three different experimental groups (F(2, 24) = 0.72355, *p* = 0.49531) (Figure 4A). There was a difference in the number of rearing acts (F(2, 24) = 7.2142, *p* = 0.00352). The largest number of rearing acts was found in the group of 2-month-old mice (33.9 ± 3). The smallest number was in the group of 15-month-old mice (14.3 ± 3.4) (differences with 2-month-old mice were statistically significant (*p* < 0.01)). This may indicate an age-related decrease in exploratory behavior (Figure 4B). No differences were found in the number of entries to the center area (F(2, 24) = 1.5954, *p* = 0.22361) (Figure 4C). The time spent by the mice in the center of the open field also did not differ in the three experimental groups (F(2, 24) = 2.5890, *p* = 0.09591) (Figure 4D). The number of grooming acts (F(2, 24) = 0.46847, *p* = 0.63156) and the grooming time (F(2, 24) = 1.6499, *p* = 0.21311) did not differ, which may indicate that the level of anxiety does not change with age (Figure 4E,F). The number of hole-poking acts was highest in the group of 2-month-old mice (5.4 ± 1.2). In 7-month-old mice, the mean number of hole-poking acts was 4.8 ± 1.2. In 15-month-old mice, the mean number of hole-poking acts was 2.1 ± 0.6, which was statistically significantly lower compared to 2-month-old mice (*p* < 0.05) and 7-month-old mice (*p* < 0.05) (Figure 4G). The number of acts of defecation did not differ between the three different experimental groups F(2, 24) = 1.7528, *p* = 0.19475 (Figure 4H).

**Figure 5:**
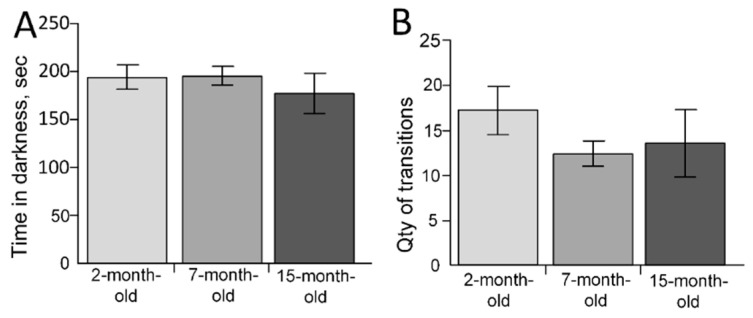
The results of the dark-light box test. (**A**) Time in the dark compartment of the box (s); (**B**) Number of transitions between compartments of the box. The 2-month-old mice (n = 9), 7-month-old mice (n = 9), 15-month-old mice (n = 9).

The DLB test showed that the time spent by mice in the dark compartment did not differ in the three age groups (F(2, 24) = 0.43954, *p* = 0.64942) (Figure 5A). There were also no differences in the number of transitions between dark and light compartments (F(2, 24) = 0.82031, *p* = 0.45227) (Figure 5B).

### 2.6. The Change of Cognitive Function

**Figure 6:**
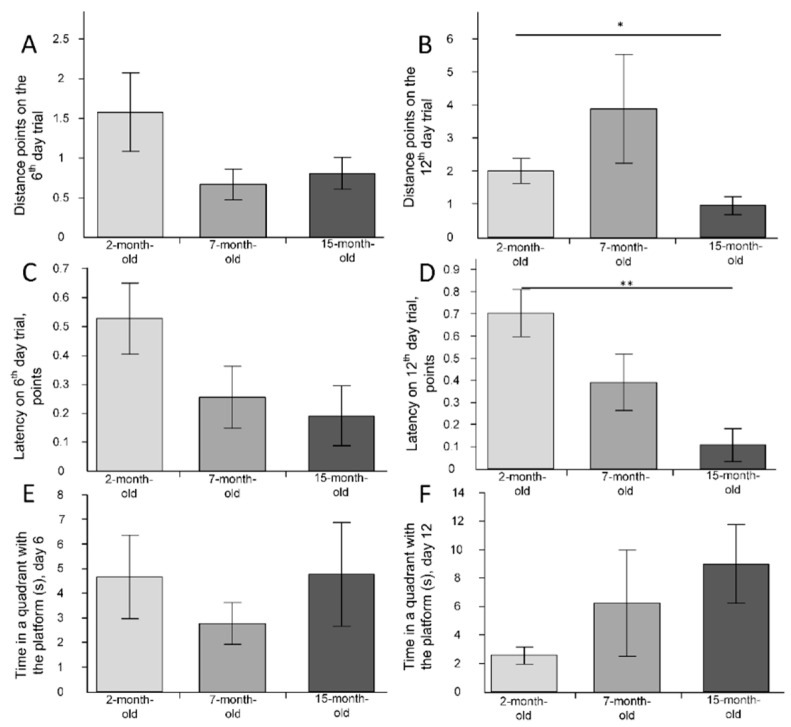
The results of the Morris water maze for reference memory. (**A**) Distance points on the sixth trial day; (**B**) distance points on the twelfth trial day; (**C**) latency time points on the sixth trial day; (**D**) latency time points on the twelfth trial day; (**E**) time in a quadrant with the platform(s) on the sixth trial day; (**F**) time in a quadrant with the platform (s) on the twelfth trial day. * *p* < 0.05; ** *p* < 0.01 differences between age groups were statistically significant according to the Mann–Whitney test. The 2-month-old mice (n = 9), 7-month-old mice (n = 9), 15-month-old mice (n = 9).

**Figure 7:**
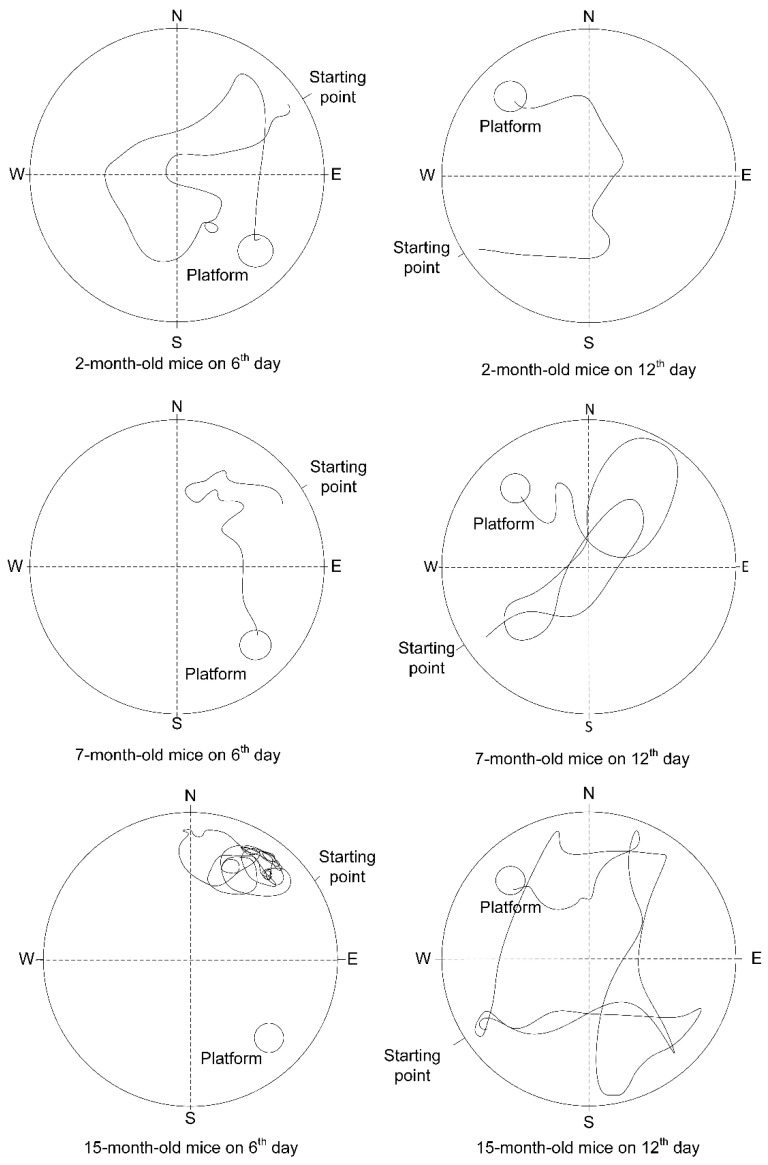
The results of the Morris water maze for reference memory. The most representative pathways for mice from 3 groups are shown on the sixth trial day (**left** column) and the twelfth trial day (**right** column).

When assessing reference memory, no differences were found in the number of points of the distances noted on the sixth day of the research (F(2, 24) = 2.2313, *p* = 0.12919) (Figure 6A). On the twelfth day of the study, the mean points obtained by animals in the group of 2-month-old mice was 2 ± 0.4, in 7-month-old animals was 3.9 ± 1.6, and in 15-month-old animals was 1 ± 0.3, which was statistically significantly less than in the 2-month-old group (*p* < 0.05) (Figure 6B). The most representative pathways for mice from the three groups are shown in Figure 7.

The number of points of latency time on the sixth day of the study did not differ in the three age groups (F(2, 24) = 2.5600, *p* = 0.09823) (Figure 6C). The latency time points on the twelfth day of the study for the group of 2-month-old mice were 7 times higher than in the group of 15-month-old mice (*p* < 0.01) (Figure 6D). The time spent in the quadrant with the goal platform on the sixth and twelfth days did not differ for animals of different ages (Figure 6E,F).

**Figure 8:**
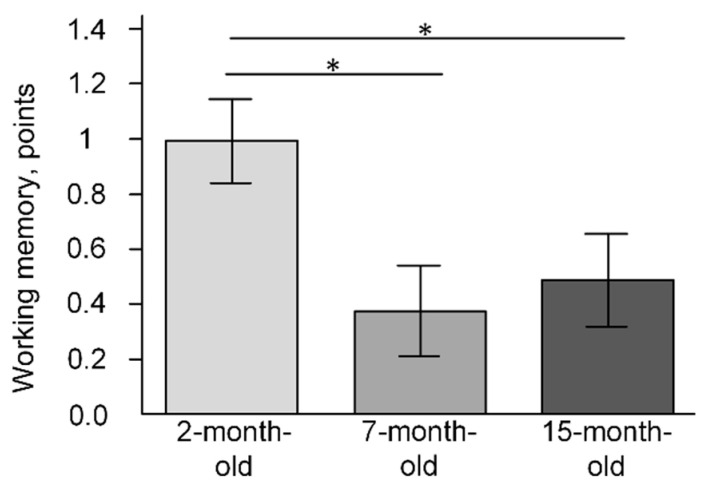
The results of the Morris water maze for working memory (points). * *p* < 0.05 differences between age groups were statistically significant according to the Mann–Whitney test. The 2-month-old mice (n = 9), 7-month-old mice (n = 9), 15-month-old mice (n = 9).

The MWM showed that the working memory of the mice was reduced by the age of 7–15 months (F(2, 23) = 3.8386, *p* = 0.03643). The mean number of points received by 2-month-old mice was 1 ± 0.2 and by 7-month-old mice was 0.4 ± 0.2, which was statistically significantly lower than in the 2-month-old group (*p* < 0.05). In the group of 15-month-old mice, the mean number of points was 0.5 ± 0.2, which was statistically significantly lower than in the 2-month-old group (*p* < 0.05) (Figure 8).

### 2.7. The Changes of Strength and Endurance

**Figure 9:**
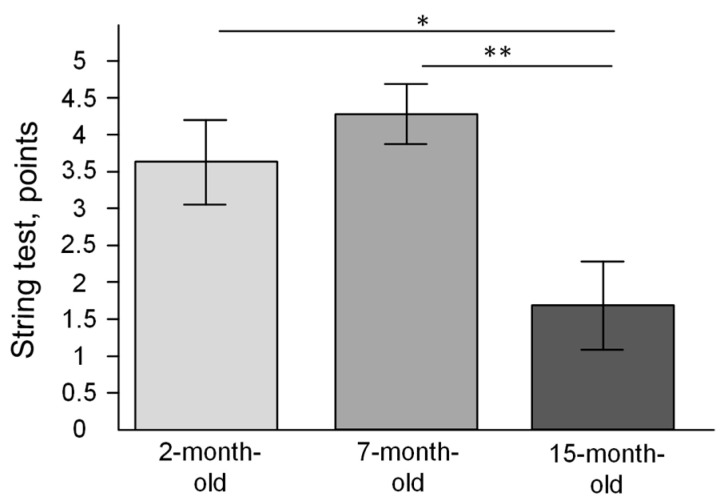
The results of the string test (scores). * *p* < 0.05; ** *p* < 0.01 differences between age groups were statistically significant according to the Mann–Whitney test. The 2-month-old mice (n = 9), 7-month-old mice (n = 9), 15-month-old mice (n = 9).

The string test showed that the strength and endurance of the mice were significantly reduced by the age of 15 months (F(2, 24) = 6.4307, *p* = 0.00580). The mean number of obtained points was 1.7 ± 0.6, which was significantly less than in 2-month-old mice (3.6 ± 0.6 points) (*p* < 0.05)) and in 7-month-old mice (4.3 ± 0.4 points) (*p* < 0.01) (Figure 9).

## 3. Discussion

The Nrf2/ARE signal pathway plays a key role in the regulation of intracellular homeostasis protection of cells by induction of expression of phase II detoxifying and oxidative-stress responsive genes. Nrf2 is inhibited by Kelch-like ECH-associated protein1 (Keap1), glycogen synthase kinase (GSK3β), and the E3 ubiquitin ligase Hrd1 [13]. Earlier, it was suggested that a decline in Nrf2 function is a critical component of the aging process [14]. We showed that *Nfe2l2* expression was dramatically decreased in the 15-month-old mice in the cerebral cortex (Figure 2D). A decrease in *Nfe2l2* is consistent with the decline of *Sod2* expression (Figure 2G), which protects mitochondrial components from superoxide-induced damage and may be positively regulated by the Nrf2/ARE signal pathway [15]. In recent years, the number of publications on the essential role of Nrf2/ARE signaling in the regulation of mitochondrial biogenesis has grown exponentially. Surprisingly, we found a negative correlation (rs = −0.44, *p* < 0.05) between the level of *Nfe2l2* expression in the cerebral cortex and copy number of mtDNA (Figure 1B), which appears surprising in light of the fact that Nrf2 should regulate mitochondrial biogenesis [5]. It is likely that, with aging, the positive feedback of the regulation of mitochondrial biogenesis is disrupted. However, we have shown that the number of mtDNA copies increases with age in the cerebral cortex (Figure 1B). Previous data on age-related changes in the number of copies of mitochondria in the brain are different and sometimes opposite. According to Neuhaus et al. (2017), the number of mtDNA copies in the adrenal cortex decreases with age [16]. An age-related decrease in the activity of NADH dehydrogenase [17,18,19] and citrate synthase [20] has also been shown. At the same time, there is evidence that cytochrome c oxidase activity increases with age [21] and increases the amount of cytochrome oxidase protein [20,22].

However, it is unlikely that the age-related increase in the number of mtDNA copies is associated with the activation of mitochondrial biogenesis. We did not find an age-related increase in the expression of the *Nrf1* and *Tfam* genes, which are responsible for mtDNA replication [23]. Mitochondrial biogenesis is not the exclusive process that regulates the mtDNA and mitochondria copy number. Mitophagy is a mitochondrial quality control mechanism that enables the degradation of damaged mitochondria [24]. We showed that expression of the *p62* gene significantly decreased with age in the cerebral cortex (Figure 2H). It is understandable since *p62* expression is also known to be regulated by the Nrf2/ARE signaling pathway [25]. P62 acts as a shuttle protein to transport polyubiquitinated protein for both lysosomal and proteasomal degradation in the autophagy and mitophagy processes [26]. Age-related mitophagy pathologies may be reasons for an increase in mtDNA copy number in the 15-month-old mice. In this case, the amount of mtDNA with a damaged base should increase. Indeed, we found that the number of mtDNA damage increased significantly in the 15-month-old mice (Figure 1A).

Thus, it is more probable that the age-related increase in the number of mtDNA copies is associated with mitophagy dysregulation but not with mitochondrial biogenesis dysfunction. In general, these processes may disrupt mitochondrial quality control in the cerebral cortex. Indeed, the MWM showed a deficit in working (Figure 8) and reference memory (Figure 6 and Figure 7) for 15-month-old-mice. It should be noted that on the sixth day of the experiment, there were no statistically significant differences in the latency time and distance that the mice swam in search of the platform (Figure 6A,C). Differences were found on the 12th day of the experiment, after moving the platform to the opposite quadrant (Figure 6B,D). Reversal learning and subsequent trial reveal whether or not animals can extinguish their initial learning of the platform’s position and acquire a direct path to the new goal position [27]. It may indicate that 15-month-old mice switch their attention to a new platform location that is significantly worse.

Early age-related deficits were shown for C57BL/6 mice repeatedly [28,29,30,31,32]. We can assume that Nrf2/ARE downregulation in the cerebral cortex may be associated with cognitive decline in aged mice. Our data confirmed that *Nrf2* had a positive effect on the oxidative status of the cell, as we found a negative correlation between the number of mtDNA lesions and the level of *Nfe2l2* expression in the cerebral cortex (r_s_ = −0.37, *p* < 0.05). The level of mtDNA damage also negatively correlated with the results of the MWM (latency on the 12th day of the experiment) (r_s_ = −0.57, *p* < 0.05). This may indicate that the functional integrity of mitochondria, in particular mtDNA, plays a key role in maintaining long-term memory. In general, it can be noted that the amount of mtDNA damage increases significantly with age, which may be a consequence of dysregulation of the Nrf2 function, as well as one of the causes of impaired cognitive function [33].

There is evidence that the transcription factor, Nrf2, also plays an important role in long-term potentiation. Nrf2 can indirectly participate in this process by maintaining the energy homeostasis of mitochondria and directly, by forming a regulatory loop with the BDNF/AKT1/mTORC1 axis [12]. The promotor region of the *Mtor* gene is contained in the ARE sequence, and therefore its expression may be regulated by Nrf2 [34]. These data indicate that the Nrf2/ARE signaling pathway must be involved in the formation of long-term memory. However, no positive correlation was found between the level of *Nfe2l2* expression in the hippocampus and points of long-term memory. Meanwhile, a positive correlation was found between the expression of *Nfe2l2* in the cerebral cortex and the point of latency on the sixth (r_s_ = 0.44, *p* < 0.05) and twelfth trial days (r_s_ = 0.44, *p* < 0.05). It is known that the cortex (especially the prefrontal cortex) is more involved in long-term memory retrieval [35]. For this reason, we can assume that Nrf2 plays an essential role in maintaining the mitochondrial homeostasis of the cortical neurons, and a high level of *Nfe2l2* expression is important for long-term memory retrieval.

It is widely known that the BDNF/AKT1/mTORC1 axis plays an essential role in regulating long-term memory formation. This process takes place mainly in the hippocampus [12]. We have shown that age-related changes in gene expression of these signaling pathways are extremely heterogeneous. The expression of *Akt1*, *Mtor*, and *Bdnf* in the hippocampus was maximal in 2-month-old mice, but in *Akt1* and *Mtor*, it was minimal in 7-month-old mice (Figure 3). At the same time, the expression of *Pten*, which is a negative regulator of the BDNF/AKT1/mTORC1 axis, on the contrary, was maximal in 7-month-old mice (Figure 3). There was a positive correlation between the expression of *Bdnf* in the hippocampus and the point of latency on the sixth day of the trial (r_s_ = 0.37, *p* < 0.05) and the twelfth day of the trial (r_s_ = 0.52, *p* < 0.05), which may confirm a relationship between the formation of long-term memory and *Bdnf* gene expression.

Another aspect of aging is the increase in inflammation [36]. We have shown that the expression of *Il1b* in the cerebral cortex significantly increases with age (Figure 2B), which may indicate a course of intense inflammatory processes [37]. At the same time, there was a pronounced negative correlation between the level of expression of the *Nfe2l2* and *Il1b* genes (r_s_ = −0.45, *p* < 0.05). This assumes that Nrf2 binds to the proximity of inflammatory cytokine genes, including IL1b, and inhibits their transcription [38]. An increase in the expression of pro-inflammatory cytokines may be one of the causes of some cognitive and behavioral deviations [39].

In general, we identified age-related deviation of physiological parameters in almost every test used. The open-field test showed an age-related decrease in the number of rearing acts (Figure 4B) and hole-poking (Figure 4G), which may indicate a decrease in exploratory behavior [40]. Malatynska et al. (2012) showed the following similar age-related changes in mice: a decrease in nose-poke behavior and a decrease in rearing were demonstrated [41]. In studies by Benice et al. (2006) [42], Lalonde and Strazielle (2009) [43], and Shoji et al. (2016) [44], it was shown that distance traveled and time spent in the center decreased with age, which may also indicate a decrease in the level of exploratory behavior and increased anxiety-related behavior [45]. However, in our study, these indicators did not change with age (Figure 4A,D). Perhaps this can be explained by the fact that Benice et al. (2006) [42], Lalond and Strazielle, (2009) [43], and Shoji et al. (2016) [44] used C57BL/6J mice, while in our work, the C57BL/6N strain was used. It is known that the C57BL/6N strain has greater fear of learning and anxiety than in C57BL/6J, whereas pain sensitivity and rotarod performance are greater in C57BL/6J than in C57BL/6N [46,47,48]. In our experiment, we used males only. It may be another reason for the lack of age-related differences in the anxiety level because it is known that males have lower levels of anxiety compared with females [49].

The differences were also shown using the DLB test. A study by Shoji et al. (2016), using the C57BL/6J strain, showed an age-related decrease in the number of transitions and time spent in the light compartment [44], while the C57BL/6N strain did not show any changes in our experiment (Figure 5) and a study by Malatynska et al. (2012), who also used the C57BL/6N strain [41]. In our study, the string test showed an age-related decrease in strength and endurance (Figure 9), while in the study of Shoji et al. (2016), there was an age-related decrease in average latency in the rotarod test, while grip strength was unchanged [44]. Thus, differences between our data and early observed results may be explained by differences between C57BL/6J and C57BL/6N mice.

## 4. Materials and Methods

### 4.1. Animals

**Figure 10:**
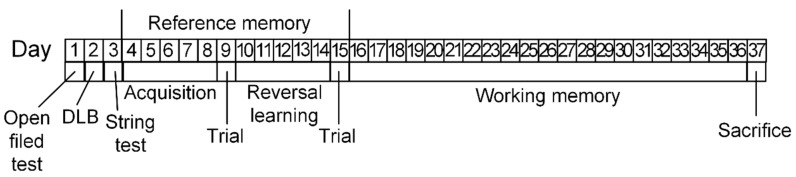
Timeline of the experiment. The open field was performed at the 1st day. The string test was performed at the 2nd day. The dark-light box (DLB) test was performed at the 3rd day. Morris water maze (MWM) for assessment of reference memory was performed from the 4th to the 15th day. MWM for assessment of working memory was performed from the 16th to the 36th day. Mice were sacrificed at the 37th day of experiment.

In total, 27 males of C57BL/6N mice, which were obtained from the Stolbovaya nursery (Russia, Moscow region), were used in the experiment. Animals were divided into three groups according to their age (2-month-old, 7-month-old, and 15-month-old) with nine animals in each group. The choice of the following age groups is based on our earlier study, where it was shown that at the age of 15 months, mice begin to show behavioral abnormalities [50]. Growing, keeping, and sacrificing animals were performed according to the rules set by the Voronezh State University Ethical Committee on Biomedical Research (Section of Animal Care and Use, protocol 42-03 dated 8 October, 2020). Animals were housed per 3 mice in a plastic cage (30 cm × 20 cm × 18 cm). Standard laboratory diet (Ssniff Spezialdiaten GmbH, Soest, Germany) and drinking water were available *ad libitum*. On the 1st day of experiment, open field test was performed. On the 2nd day, the dark-light box test was performed. On the 3rd day, the string test was performed. From the 4th to 36th day of experiment, Morris water maze was performed. Timeline of experiment is presented in Figure 10. After the physiological experiments, mice were sacrificed by anesthetization with a mixture (1 mL/kg) of xylazine (10 mg/kg) and ketamine (90 mg/kg) administered intraperitoneally. After decapitation, the brain was removed and dissected. The cerebral cortex and hippocampus tissues [51] were immediately frozen at −80 °C and subsequently used for RNA and DNA extraction.

### 4.2. The Open Field Test

The animal was placed in the open arena (40 × 40 × 60 cm) with five randomly distributed holes (0.5 cm diameter). Lighting conditions were set to 100 lux. The arena was a square white box marked with square crossings on the floor to determine the position of the animal. Center of arena was square 20 × 20 cm. The new environment could cause the following two types of reactions: exploratory behavior and defensive behavior caused by fear.

In this experiment, we evaluated such indicators as locomotor activity (s), activity in the center of area (s), the number of exits to the center of area, the level of hole poking, the number of rearing acts, defecation acts, and the duration (s) and quantity of grooming acts. The grooming act was completed if the mouse touched any part of its body by nose and performed the grooming act at least a second. If the mouse paused for 3 s or more, then this was considered the next act of grooming. The duration of the experiment was 5 min [50].

### 4.3. The String Test

The mouse, holding its forelimbs, was suspended on a string (wire 3 mm diameter and 50 cm long) at a height of 50 cm above the soft surface. Foam supports for string were located along the edges of the string. The state of the animal was assessed at points after 1 min according to the following scheme: 0 = mouse fell; 1 = the mouse holds on to the string with two forelimbs; 2 = holds the string with two forelimbs and moves along the string; 3 = two forelimbs + one or two hind limbs; 4 = four limbs and a tail around the string; 5 = escape from the string to the foam support; more than five points were assigned if the escape faster than 60 s (e.g., 5.5 = exit to the end of the string in 30 s) [52].

### 4.4. The Dark-Light Box Test

DLB consisted of two compartments. The light compartment (24 × 20 × 25 cm) took up 2/3 of the test system and did not have a lid (lighting conditions were set to 100 lux), while the dark compartment (12 × 20 × 25 cm) took up 1/3 and was covered with a lid on top. There was a small hole (door 4 × 5 cm) between the compartments for the free moving. At the beginning of the experiment, the animal was placed in the light compartment and had the ability to move freely between the dark and light parts of the chamber for 5 min. The time spent in the light or dark part of the chamber, as well as the number of transitions between compartments, served as an indicator to assess the anxiety of the animal. All four paws of the animal should be located in the opposite part of the chamber so that the transition was considered completed [53].

### 4.5. The Morris Water Maze Test

To study reference and working memory of the mice, the MWM was used [27]. The MWM was conducted in a round arena (diameter 150 cm) with a platform in the target quadrant. The arena was filled with water so that the platform was hidden below the water’s surface (0.5 cm). The water was colored with titanium dioxide so that the mouse could not see the bottom. The water temperature was 25 °C. The study of reference memory took place in two stages. In the first stage (acquisition), the animals were trained for five days to reach the platform in under 1 min. The animal must learn to use distal cues (squares, circles, and triangles cut from cardboard) to navigate a direct path to the platform when starting from different locations around the perimeter of the arena. To assess reference memory at the end of learning on the 6th day, a probe trial is given. Each animal was given one trial to reach the platform in under 1 min. The second stage (reversal) of the study of reference memory included the same stages as the first, but platform was located in the opposite quadrant. Relocation platform in the MWM reveals whether or not animals can extinguish their initial learning of the platform’s position and acquire a direct path to the new goal position. Reversal learning was conducted from 7th to 11th day of experiment. On the 12th day, each animal was given one trial to reach the platform in under 1 min. The scheme of location of starting point and goal platform are presented in Table 2. The following parameters were evaluated: latency (points) to reach the goal; distance from the starting point to the platform (points); the time the animal spent in the goal quadrant (s) were measured to study reference memory. Latency points were calculated as points = 60 − latency time (s)/60. Distance points were calculated as points = (1/distance (cm)) × 1000.

To study working memory, the animal was given two attempts to find the platform (interval was 15 s). In this case, the difference in latency(s) to reach the platform was an important indicator. The scheme of the location of the starting point and goal platform are presented in Table 3. The study lasted 21 days. Tracking the animals from the starting point to the platform was carried out using the ToxTrac software [54].

### 4.6. DNA and RNA Extraction

Total DNA from brain compartment evaluation was performed with a ProbaGS kit (DNA-Technology, Moscow, Russia) according to its protocol. To extract RNA from the sample, a commercial ExtractRNA kit (Evrogen, Moscow, Russia) was used according to its protocol.

### 4.7. Reverse Transcription

For reverse transcription, the “Eppendorf Mastercycler personal” instrument was used. In total, 5 μg of RNA was mixed with 0.5 μg of oligo (dT) and deionized water, and incubated for 5 min at 70 °C. Further 2 μL of 10× buffer (SybEnzyme^®^, Moscow, Russia) and 2 μL of 10 mM dNTP, M-MuLV reverse transcriptase (SybEnzyme^®^) (40 units) were used for the reverse transcription reaction. Incubation continued for 60 min at 37 °C.

### 4.8. Quantitative PCR Analysis

The Bio-Rad CFX96^TM^ Real-Time System was used for quantitative PCR analysis. The reaction mixture contained 5 μL qPCRmix-HS SYBR + ROX; 20 pM primer combination (forward and reverse); 10 ng DNA template. The *18s* rRNA gene and *Gapdh* (glyceraldehyde-3-phosphate dehydrogenase) were used as the reference genes. The primer sequences are presented in Table 4.

Total denaturation was observed at 95 °C for 3 min; denaturation at the beginning of the cycle at 95 °C for 30 s; primer annealing at 61 °C for 30 s, elongation at 72 °C for 30 s; number of cycles 38; total elongation 72 °C for 5 min; melting curve from 65 °C to 95 °C. Quantitatively normalized expression of genes was expressed as relative single fluorescence.

### 4.9. Measurement of mtDNA Damage

The number of mtDNA damage was evaluated by long-range PCR using Encyclo polymerase (Evrogen, Russia) on a CFX96TM Real-Time System thermal cycler (Bio-Rad, Hercules, CA, USA), according to the protocol described by Gureev et al. (2017) [55]. The method of measurement of mtDNA damage by long-range PCR based on the assumption that DNA damage such as single-strand breaks slows down accumulation of the PCR product due to the inhibition of elongation process. Therefore, the rate of PCR product accumulation would be inversely proportional to the number of damaged DNA molecules [55]. Measurement of damage number was carried out in the six long fragments of mtDNA (NC_005089.1) [56]. In this research we used mtDNA fragments, which were not duplicated in the nuclear genome. Primer sequences were present in Table 5.

The ΔCq value of the control and experimental long fragments were compared with the ΔCq of the control and experimental short fragments. Short fragments used as references for normalization of mtDNA copy number. The amount of additional damage in mtDNA was calculated at 10 kb according to the following formula:

Damage = (1 − 2^−(Δ*long* − Δ*short*)^) × (10,000 (bp)/length of the fragment (bp))

### 4.10. MtDNA Copy Measurement

The amount of mtDNA was evaluated by quantitative PCR. For this, amplification of mtDNA fragments was performed using the following primers:

F: 5′-ACGAGGGTCCAACTGTCTCTTA-3′; R: 5′-AGCTCCATAGGGTCTTCTCGT-3′.

The nuclear DNA encoded *Gapdh* gene was used as a reference. The primer sequences were as follows:

F: 5′-GGCTCCCTAGGCCCCTCCTG-3′; R: 5′-TCCCAACTCGGCCCCCAACA-3′.

The normalized level of mtDNA relative to nuclear DNA was calculated by the following formula: 2^(−ΔΔCq).^

### 4.11. Statistical Analysis

Statistical analysis was performed using the Statistica 10.0 software package (StatSoft., Inc., Tulsa, OK, USA). The normality of data distribution was determined using the Shapiro–Wilk test. The result is presented as the mean ± SEM. The results were analyzed by one-way analysis of variance (ANOVA). Tukey’s post-hoc test was used to determine the significance level. Correlation analysis was performed using Spearman’s rank correlation coefficient (r_s_). For cluster analysis, a hierarchical algorithm (single-linkage clustering) was used. The Euclidean distance and 1 – Pearson R were used for measuring distances between groups of variables.

## 5. Conclusions

Thus, we showed that the Nrf2/ARE signal pathway was downregulated in the cerebral cortex of 15-month-old mice. Despite the fact that Nrf2 is involved in the regulation of mitochondrial biogenesis, we showed an increase in the mtDNA copy number in aged mice. Possibly, it is associated with the downregulation of mitophagy, which is also under Nrf2 control. As a result, damaged mtDNA accumulated in the cells of the cerebral cortex in the 15-month-old mice. In the hippocampus, we did not find an age-related decrease in Nrf2 signaling, but *Bdnf* suppression may be associated with the cognitive and behavioral decline of aged mice.

## Figures and Tables

**Table 1:** Number of mtDNA lesions in the cerebral cortex in the three age groups of mice.

Fragment	Number of mtDNA Lesions (per 10 kb)		
	2-Month-Old	7-Month-Old	15-Month-Old
12–16s (1st)	2.36 ± 0.39	4.01 ± 0.37 **	3.34 ± 0.30
*16s-Nd1* (2nd)	2.45 ± 0.41	3.59 ± 0.71	4.11 ± 0.29 *
*Nd1-Nd2* (3th)	1.03 ± 0.36	2.86 ± 0.62 *	2.05 ± 0.15
*Nd5* (4th)	1.97 ± 0.43	3.12 ± 0.51	2.65 ± 0.11
*Nd6-CytB* (5th)	2.16 ± 0.34	1.89 ± 0.59	2.73 ± 0.19
*D-loop* (6th)	3.89 ± 0.54	3.02 ± 0.67	5.44 ± 0.07 ***^††^

* *p* < 0.05; ** *p* < 0.01; *** *p* < 0.001 differences are statistically significant compared with the 2-month-old mice group. ^††^ *p* < 0.01 differences are statistically significant for the 15-month-old compared with the 7-month-old mice group. The 2-month-old mice (n = 9), 7-month-old mice (n = 9), 15-month-old mice (n = 9).

**Table 2:** Scheme of Morris water maze experiment for the study of reference memory with an indication of starting point and goal platform.

Acquisition. Start Located in the SW				
Day	Trial 1	Trial 2	Trial 3	Trial 4
1	N	E	SE	NW
2	SE	N	NW	E
3	NW	SE	E	N
4	E	NW	N	SE
5	N	SE	E	NW
6 (Probe)	NE	-	-	-
**Reversal. Start Located in the NE**				
Day	Trial 1	Trial 2	Trial 3	Trial 4
1	S	W	NW	SE
2	NW	S	SE	W
3	SE	NW	W	S
4	W	SE	S	NW
5	S	NW	W	SE
6 (Probe)	SW	-	-	-

**Table 3:** Scheme of Morris water maze experiment for the study of working memory with an indication of starting point and goal platform.

Day	Start	Goal	Day	Start	Goal
1	N	SE	11	E	SW
2	E	NE	12	N	SW
3	S	SW	13	E	NW
4	W	SE	14	W	NE
5	S	NE	15	N	SE
6	N	NW	16	S	SW
7	W	NE	17	N	NE
8	E	SE	18	S	NW
9	W	NW	19	E	NW
10	S	SE	20	W	SW
			21	N	SE

**Table 4:** Primer sequences for gene expression assessment.

Gene Name	Transcript Accession Number	Forward Primer 5′–3′	Reverse Primer 5′–3′
*18s*	NR_003278.3	CGGCTACCACATCCAAGGAA	GCTGGAATTACTGTGGCT
*Gapdh*	NC_000072.7	GGCTCCCTAGGCCCCTCCTG	TCCCAACTCGGCCCCCAACA
*Nfe2l2*	NM_010902.4	CTCTCTGAACTCCTGGACGG	GGGTCTCCGTAAATGGAAG
*Bdnf*	NM_007540.4	AAGGACGCGGACTTGTACAC	CGCTAATACTGTCACACACGC
*Mtor*	NM_020009.2	AGATAAGCTCACTGGTCGGG	GTGGTTTTCCAGGCCTCAGT
*Akt1*	NM_009652.3	TGATCAAGATGACAGCATGGAGTG	GATGATCCATGCGGGGCTT
*Pten*	NM_008960.2	AGGGACGAACTGGTGTAATGA	GGGAATAGTTACTCCCTTTTTGTCT
*Il1b*	NM_008361.4	TTGACGGACCCCAAAAGATG	AGAAGGTGCTCATGTCCTCA
*Nrf1*	NM_001164226.1	AGCACGGAGTGACCCAAA	TGTACGTGGCTACATGGACCT
*Cox1*	NC_005089.1	TCGCAATTCCTACCGGTCTC	CGTGTAGGGTTGCAAGTCAGC
*p62*	U17961.1	GCCAGAGGAACAGATGGAGT	TCCGATTCTGGCATCTGTAG
*Sod2*	NM_013671.3	CAGACCTGCCTTACGACTATGG	CTCGGTGGCGTTGAGATTGTT
*Tfam*	NC_000076.7	ATTCCGAAGTGTTTTTCCAGCA	TCTGAAAGTTTTCGATCTGGGT

**Table 5:** Primer sequences for measurement of mtDNA damage.

Fragment Name	Forward Primer 5′–3′	Reverse Primer 5′–3′	Length Fragment
Short	ACGAGGGTCCAACTGTCTCTTA	AGCTCCATAGGGTCTTCTCGT	97 bp
*12–16s (1st)*	TAAATTTCGTGCCAGCCACC	ATGCTACCTTTGCACGGTCA	1739 bp
*16s-Nd1 (2nd)*	ACGAGGGTCCAACTGTCTCTTA	CCGGCTGCGTATTCTACGTT	1326 bp
*Nd1-Nd2 (3th)*	CTAGCAGAAACAAACCGGGC	TTAGGGCTTTGAAGGCTCGC	1675 bp
*Nd5 (4th)*	TCATTCTTCTACTATCCCCAATCC	TGGTTTGGGAGATTGGTTGATG	1942 bp
*Nd6-CytB (5th)*	CCCCAATCCCTCCTTCCAAC	GGTGGGGAGTAGCTCCTTCTT	1732 bp
*D-loop (6th)*	AAGAAGGAGCTACTCCCCACC	GTTGACACGTTTTACGCCGA	1308 bp

## Data Availability

Not applicable.
